# Activation of Nuclear Factor Kappa B in the Hepatic Stellate Cells of Mice with Schistosomiasis Japonica

**DOI:** 10.1371/journal.pone.0104323

**Published:** 2014-08-12

**Authors:** Xing He, Guangbin Pu, Rui Tang, Dongmei Zhang, Weiqing Pan

**Affiliations:** Department of Tropical Infectious Diseases, Second Military Medical University, Shanghai, China; Bambino Gesu' Children Hospital, Italy

## Abstract

Schistosomiasis japonica is a serious tropical parasitic disease in humans, which causes inflammation and fibrosis of the liver. Hepatic stellate cells (HSCs) are known to play an important role in schistosome-induced fibrosis, but their role in schistosome-induced inflammation is still largely unknown. Here, we use a murine model of schistosomiasis japonica to investigate the role that nuclear factor kappa B (NF-κB), a critical mediator of inflammatory responses, plays in schistosome-induced inflammation. We revealed that NF-κB was significantly activated in HSCs at the early stage of infection, but not at later stages. We also show that the expression levels of several chemokines regulated by NF-κB signaling (Ccl2, Ccl3 and Ccl5) were similarly elevated at early infection. TLR4 signaling, one of the strongest known inducers of NF-κB activation, seemed not activated in HSCs post-infection. Importantly, we found that levels of miR-146 (a known negative regulator of NF-κB signaling) in HSCs opposed those of NF-κB signaling, elevating at later stage of infection. These results indicate that HSCs might play an important role in the progression of hepatic schistosomiasis japonica by linking liver inflammation to fibrosis via NF-κB signaling. Moreover, our work suggests that miR-146 appeared to regulate this process. These findings are significant and imply that manipulating the function of HSCs by targeting either NF-κB signaling or miR-146 expression may provide a novel method of treating hepatic schistosomiasis japonica.

## Introduction

Schistosomiasis is a serious, yet neglected, tropical parasitic disease that affects more than 200 million people worldwide [1]. Mortality from schistosomiasis occurs as a result of the development of hepatic granulomas and fibrosis, which eventually results in portal hypertension and variceal bleeding [1]. Treatment of hepatic schistosomiasis is lacking because the exact cellular and molecular mechanisms of infection and pathogenesis remain elusive. Studies have shown that the activation of hepatic stellate cells (HSCs) is central to the development of liver fibrosis from other means (including via viral infection, autoimmune deficiencies, and dietary or chemical causes) [2]. Quiescent HSCs store vitamin A in normal liver tissue, but are activated to become proliferative, contractile, and fibrogenic myofibroblasts during liver fibrosis [2], [3]. Activated HSCs secrete excess extracellular matrix (ECM), which is deposited in the liver tissue leading to fibrosis. HSCs can also function as immune cells [4], [5] with an important role in linking hepatic inflammation to fibrogenesis [6]. Importantly, accumulating evidence from both murine and human schistosomiasis reveals that HSCs also function in the granulomatous, fibrotic process induced by schistosome eggs [3], [7]. Besides, it is intriguing that a recent study showed that *Schistosoma japonicum* (*S. japonicum*) eggs induced a pro-inflammatory but anti-fibrogenic phenotype in a human HSC cell line (LX-2) [8].

The transcription factors of nuclear factor kappa B (NF-κB) family are key regulators of immune and inflammatory processes [9], [10]. When latent, NF-κB is sequestered in the cytoplasm; when stimulated by Toll-like receptors (TLRs), however, a series of membrane-proximal events leads to the nuclear translocation of NF-κB. This process subsequently, activates the transcription of many pro-inflammatory genes [9]. One critical component of the canonical NF-κB pathway is p65, also known as RelA, which can bind as a homo- or heterodimer to target DNA sequences [11]. The detection of p65 in the cell compartment is a convenient way to determine if the NF-κB pathway is activated. In addition, Seki *et al.* recently demonstrated that the NF-κB pathway was involved in the activation of HSCs via TLR4 signaling [6].

MicroRNAs (miRNAs) are a class of highly conserved, small, noncoding RNA molecules that control the translation and transcription of many genes [12], [13]. Numerous studies have revealed that miRNA plays an important role in the initiation and progression of human diseases [14], [15] and many other physiological processes [16], [17], such as immune responses, cell proliferation, cell death, and inflammation. As inflammation is also known to be regulated by NF-κB [10], many researchers have begun to examine the convergence of miRNAs and their target genes with NF-κB signaling cascades. To date, several miRNAs have been shown to be involved in the regulation of NF-κB signaling [18]. Besides, it is well acknowledged that the aberrant expression of miRNAs is associated with the pathogenesis and progression of many diseases, including liver ones [19]. It has been reported that miRNAs may play a variety of regulatory roles in the immune responses during the development of hepatic pathology after infection with schistosoma [20], [21], and some deregulated serum miRNAs can serve as potential markers for detection of schistosome infection and evaluation of the effectiveness of chemotherapy [22].

In this study, we use a well-studied murine model of schistosomiasis japonica to investigate the pro-inflammatory role of HSCs in the progression of hepatic schistosomiasis by analyzing the characteristics and regulation of NF-κB signaling. We report that HSCs appear to play an important role in linking the process of hepatic granulomatous to hepatic fibrosis via NF-κB signaling, with miR-146 potentially modulating this process by targeting TRAF6, a key adapter molecules in the TLR4/NF-κB pathway.

## Materials and Methods

### Ethics statement

This study was carried out in strict accordance with the Regulations for the Administration of Affairs Concerning Experimental Animals (approved by the State Council of the People's Republic of China) and Guide for the Care and Use of Laboratory Animals (Experimental animal center, Second Military Medical University, certificated by Shanghai Committee of Science and Technology). The protocol involving animal experiments was conducted by the approval of the Animal Ethics Committee of Second Military Medical University (laboratory animal usage number FYXK (Shanghai) 2012-0003). All animal surgery was performed under sodium pentobarbital anesthesia, and all efforts were made to minimize their pain and discomfort.

### Reagents

Anti-p65 antibody was purchased from Cell signaling (Cat. #8242, USA), and anti-TRAF6 antibody was obtained from Abcam (Cat. 33915, UK). PrimerScript 1^st^ strand cDNA synthesis Kit (Cat. 6210) and SYBR Green Master Mix kit (Cat. RR420) were purchased from Takara (Japan).

### Animals

Six-week-old male BABL/c mice were purchased from the experimental animal center of the Second Military Medical University. Mice were housed under specific pathogen-free conditions and fed autoclaved food and water as needed. Besides, mice were maintained under environmentally controlled conditions (temperature 24°C, 12 h/12 h light-dark cycle, 45% humidity).

### Schistosome infection and sample preparation

For construction of animal model of schistosomiasis, BABL/c mice were exposed percutaneously to 16 *S. japonicum* cercaria that were shed from lab-infected snails (*Oncomelania hupensis*) obtained from the National Institute of Parasitic Disease, Chinese Center for Disease Control and Prevention.

For collection of blood serum and liver samples, 20 mice were infected and randomly allocated to four groups which were euthanized on days 0, 21, 32, 42 post-infection, respectively. At each time point, five mice from the same group were fasted for twelve hours before anesthetization by intraperitoneal injection of sodium pentobarbital. After opening the abdominal cavity, blood was sampled from portal vein and inferior vena in collect serum tubes (pyrogen-free), which were then centrifuged for 10 min at 1000×g 4°C to obtain serum, which was stored at −80°C. After blood collection, the mice were killed using cervical dislocation and the liver was dissected, snap frozen in liquid nitrogen and stored at −80°C. Samples collected on day 0 after infection were considered as the control samples.

For collection of HSC samples, another 20 mice were infected and also randomly allocated to four groups which were euthanized on days 0, 21, 32, 42 post-infection, respectively. At each time point, five mice from the same group were fasted for twelve hours before anesthetization. After digested *in situ* with collagenase, livers were dissected and the mice were killed using cervical dislocation. HSCs isolated from the same time point were equally divided into three parts for total RNA, total protein, nuclear and cytoplasm protein extraction, respectively. Samples collected on day 0 after infection were considered as the control samples.

### Egg and parasite counting

The number of schistosome eggs in the liver was counted after the liver tissue was digested by 4% potassium hydroxide (KOH) [23]. Liver egg burdens were expressed as 10^4^ eggs per gram of liver tissue. Perfusions of hepatic portal system were performed to detect the number of worm pairs as described [24].

### Liver pathology

Liver specimens were fixed in 5% (v/v) paraformaldehyde in PBS. Hepatic granuloma and fibrosis were analyzed on H&E staining and Masson's trichrome staining of liver sections.

### Immunohistochemistry (ISH)

Immunoperoxidase staining was performed on 5% (v/v) formaldehyde-fixed, paraffin-embedded mouse liver sections. After routine hydration, liver sections were incubated with rabbit anti-p65 antibody for 1 hour at 37°C, and HRP-conjugated goat anti-rabbit secondary antibody (Abcam, Cat. Ab6721, UK) was used to display the signals. The number of p65 cytoplasm or nuclear positive nonparenchymal cells (NPCs), including the HSC, Kupffer cell and endothelial cell, were counted in 10 randomly chosen high-power (400×) fields in each sample. Only morphologically recognizable NPCs were counted, and the hepatocyte or infiltrated immune cell labeling was not considered. The activity of NF-κB in NPCs was calculated as the following formula: (the number of nuclear positive cell/the number of cytoplasm positive cell)×100%.

### Isolation of mouse hepatocyte, HSC, Kupffer cell and endothelial cell

Liver samples were initially digested *in situ* with 0.04% collagenase type IV. They were then further digested with 0.08% collagenase type IV while shaken for 30 minutes in a 37°C bath. The resulting cell suspension was centrifuged at 50×*g* for 4 minutes to isolate hepatocytes. Hepatocytes were further purified by centrifugation at 20×g for 4 minutes. After hepatocytes were pelleted, the supernatant containing nonparenchymal cells was further centrifuged at 500×*g* for 5 minutes. HSCs were isolated from nonparenchymal cells using 11.5% (w/v) iodixanal gradient (OptiPrep; Axis-Shield PoC AS, Oslo, Norway) at 1400×*g* for 20 minutes. To further purify HSCs, we depleted HSCs of Kupffer cells by magnetic antibody cell sorting (MACS, MiltenyiBiotec) with CD11b-conjugated microbeads (MiltenyiBiotec). The purity of HSCs was detected by flow cytometric analysis for CD11b, and real-time PCR analysis for albumin (ALB) and CD31. Kuppfer cells were first separated from nonparenchymal cells by centrifugation on a 25%–50% Percoll gradient at 900×*g* for 20 minutes. The Kupffer-cell fraction located at the interface of the 25%–50% Percoll layer. CD11b positive cells (referred to as Kupffer cells in this study) were then purified using positive selection with magnetic CD11b antibody beads and magnetic columns (MACS, Miltenyi, Auburn, CA). Hepatic endothelial cells were isolated as previously described [25].

There was no difference in the method for cell isolation from normal livers. Besides, hepatocyte, Kupffer cell and endothelial cell were isolated merely from the two groups of mice of which the infection time is 0 and 42 days.

### RNA extraction and real-time PCR

Total RNA of HSCs isolated from the four groups of mice was harvested using TRIzol (Invitrogen) according to manufacturer's instructions. The RT qPCR was performed as described previously [26]. The expression of ALB, CD31, p65, Ccl2, Ccl3, Ccl5, Cxcl1, Col1α1, α-SMA, miR-146a and miR-146b was determined using the SYBR Green Master Mix kit. GAPDH was used as an internal control for mRNA quantification while U6 snRNAs was used as an internal control for miRNA quantification, and the fold change was calculated by the 2^−ΔΔCt^ method. The sequences of the primers were shown in Table 1.

**Table 1:** Primers used in this study.

Gene	primer sequence (5′→3′)	
ALB	forward primer	CAAGAGTGAGATCGCCCATCG
	reverse primer	TTACTTCCTGCACTAATTTGGCA
CD31	forward primer	CTTCACCATCCAGAAGGAAGAGAC
	reverse primer	CACTGGTATTCCATGTCTCTGGTG
p65	forward primer	AGGCTTCTGGGCCTTATGTG
	reverse primer	TGCTTCTCTCGCCAGGAATAC
Ccl2	forward primer	TTAAAAACCTGGATCGGAACCAA
	reverse primer	GCATTAGCTTCAGATTTACGGGT
Ccl3	forward primer	TTCTCTGTACCATGACACTCTGC
	reverse primer	CGTGGAATCTTCCGGCTGTAG
Ccl5	forward primer	GCTGCTTTGCCTACCTCTCC
	reverse primer	TCGAGTGACAAACACGACTGC
Cxcl1	forward primer	ACTGCACCCAAACCGAAGTC
	reverse primer	TGGGGACACCTTTTAGCATCTT
α-SMA	forward primer	CGCTGCTCCAGCTATGTGTGA
	reverse primer	TTTGGCCCATTCCAACCATTAC
Col1α1	forward primer	GCACGAGTCA CACCGGAAC
	reverse primer	CCAATGTCCAAGGGAGCCAC
GAPDH	forward primer	ACCACAGTCCATGCCATCAC
	reverse primer	TCCACCACCCTGTTGCTGTA
miR-146a	forward primer	ATGGTTCGTGGGTGAGAACTGAATTCCA
	reverse primer	GCAGGGTCCGAGGTATTC
miR-146b	forward primer	ATGGTTCGTGGGTGAGAACTGAATTCCA
	reverse primer	GCAGGGTCCGAGGTATTC
U6	forward primer	GCTTCGGCAGCACATATACTAAAAT
	reverse primer	CGCTTCACGAATTTGCGTGTCAT

### Flow cytometry

HSCs isolated from mice after 0 or 42 days infection were incubated with FITC-conjugated anti-CD11b monoclonal antibody (mAb) (Biolegend) for 15 minutes at 4°C. The cells were analyzed on a FACS Calibur flow cytometer (BD Bioscience). HSCs were defined as cells that were CD11b^−^. Data were analyzed in Flowjo.

### Detection of LPS level in serum

LPS levels in serum obtained from the four groups of mice were detected using a color metric assay according to manufacturer's instructions (Chinese Horseshoe Crab Reagent Manufactory Co., Ltd., China). The minimum detectable concentration of kit was 0.01 EU/mL.

### Western blotting

Cell protein was extracted on ice with nuclear and cytoplasmic protein extraction kit (Beyotime) or RIPA lysis buffer in the presence of freshly added protease inhibitors (Thermo), and quantified by the BCA method (Pierce). A total of 30 µg/lane protein extract was loaded onto a 12% SDS-polyacrylamide gel and transferred to 0.2 µm nitrocellulose membranes (Pierce) with wet western blotting system for 1 hour at 350 mA. Nonspecific binding was blocked with 5% nonfat milk in TBST. The membrane was incubated with rabbit anti-p65, anti-TRAF6 antibody overnight at 4°C. IRDye 800CW goat anti-rabbit IgG (LI-COR, Cat. 926-32211) was used as secondary antibody, and rabbit anti-GAPDH (EPITOMICS, Cat. 2251-1) or anti-Histone H1.0 (EPITOMICS, Cat. 6601-1) antibody was used as an internal standard.

The activity of NF-κB was calculated as the following formula: (the intensity of nuclear p65/the intensity of nuclear Histone H1.0)/(the intensity of cytoplasm p65/the intensity of cytoplasm GAPDH).

### Statistical analyses

All results are reported as means ± standard deviations and compared between groups using two-tailed Student's *t*-test or one-way ANOVA. Data were considered statistically significant at *P*<0.05.

## Results

### Liver pathology in mice post-infection

**Figure 1:**
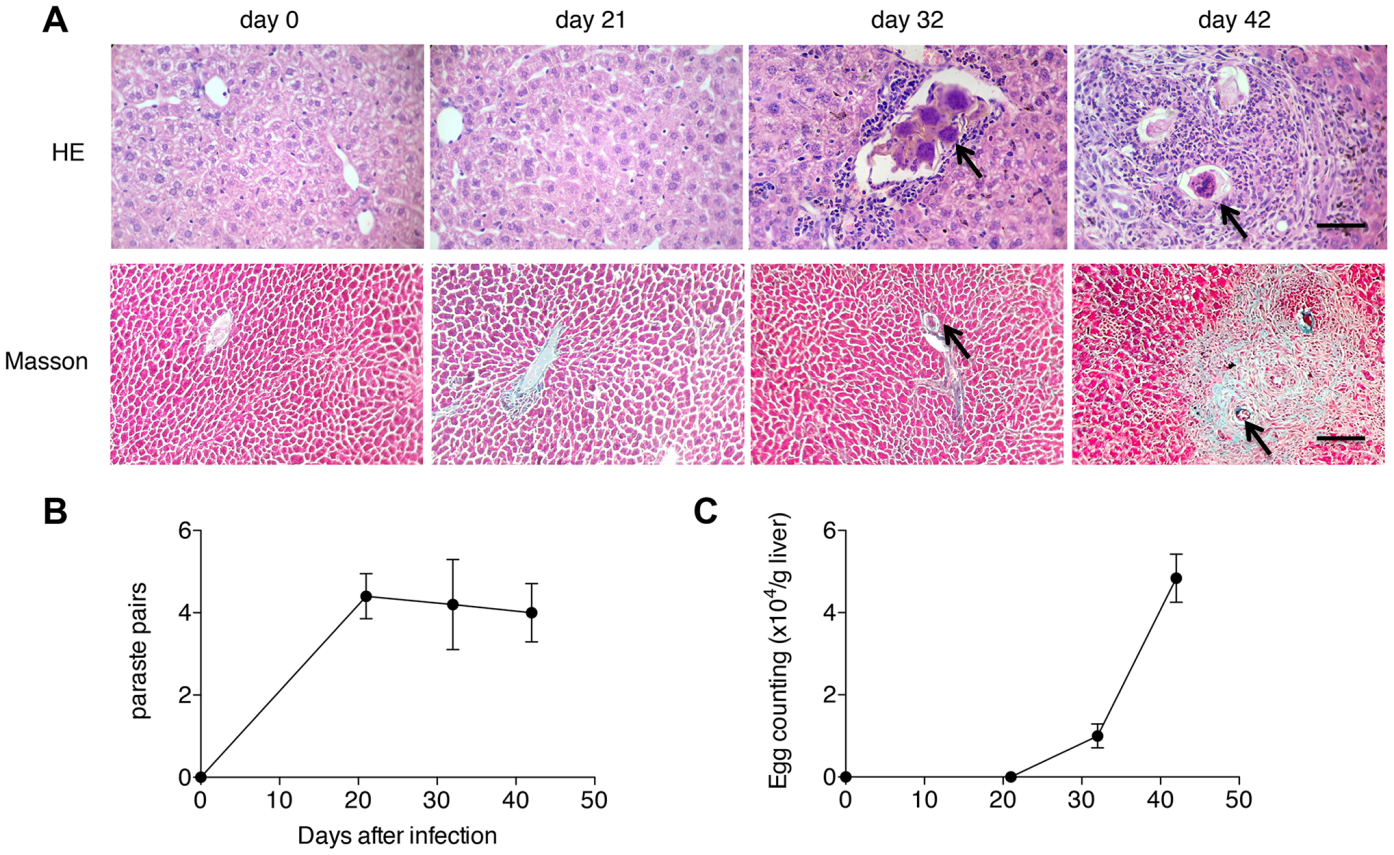
Liver pathology in the progression of hepatic schistosomiasis. (A) H&E and Masson's trichrome staining of liver sections. ECMs are shown in blue in the Masson's trichrome staining. The arrows point to schistosome eggs. Scale bar indicates 100 µm. (B) Worm pairs. (C) Egg burdens in host livers.

In order to evaluate the liver pathology in the process of hepatic schistosomiasis, mice were infected with 16 cercaria of *S. japonicum* and euthanized on days 0, 21, 32, 42 post-infection to harvest the liver samples. The results of liver section staining showed that eggs began to lodge in the liver with only minimal cellular infiltration on day 32 post-infection, while by 6 weeks post-infection, eggs were surrounded by a dense population of immune cells, leading to the formation of a mature granuloma with mild ECM deposition (Fig. 1A). We also found that although parasite burdens in the animals did not change post-infection (Fig. 1B), the number of eggs trapped in liver tissues dramatically increased after 32 days post-infection (Fig. 1C).

### The activity of NF-κB signaling in the progression of hepatic schistosomiasis

**Figure 2:**
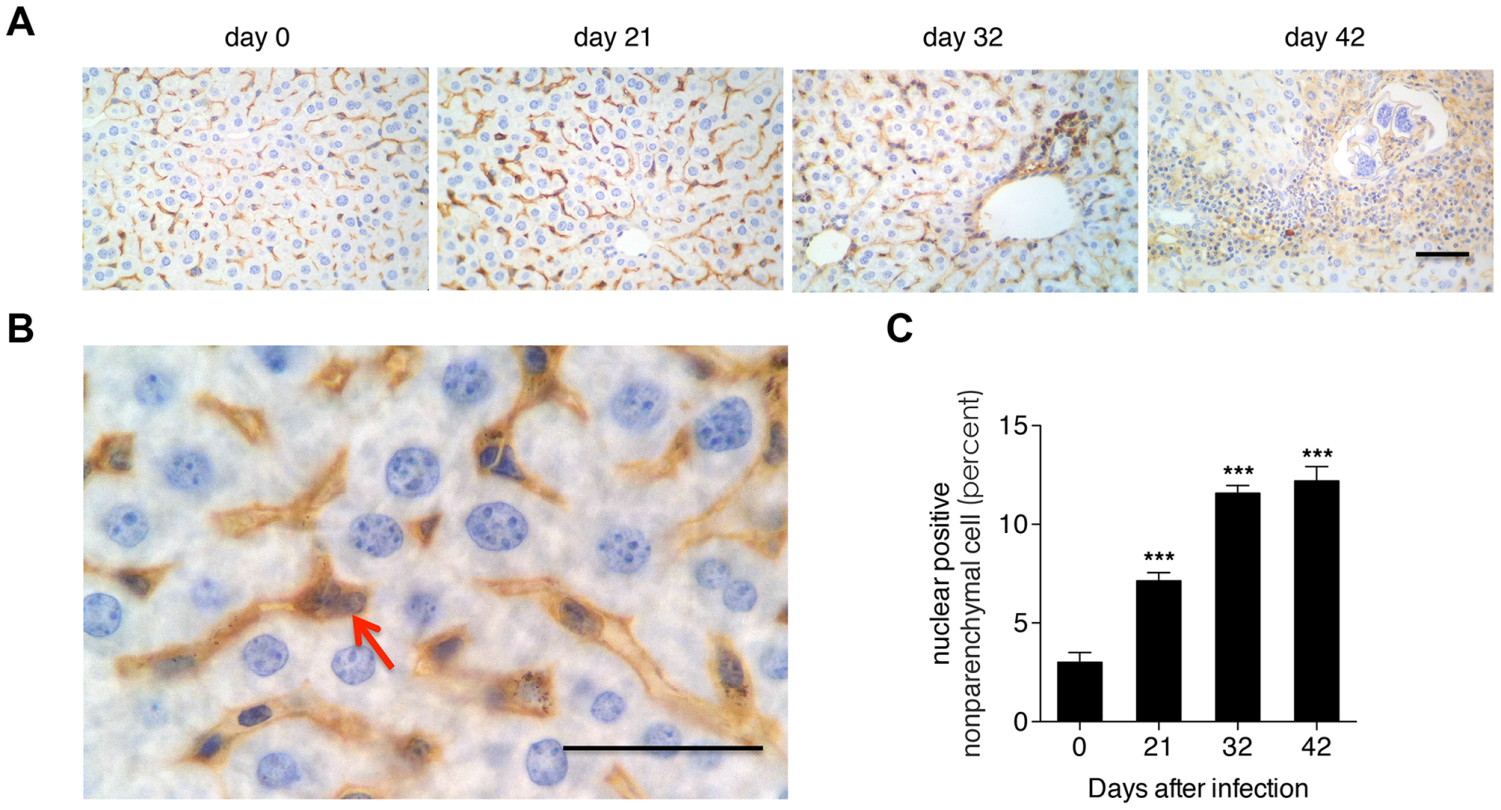
Activation of NF-κB in the nonparenchymal cells of the liver post-infection. (A) NF-κB p65 subunit immunohistochemistry of liver sections. (B) Close-up of A with the arrow indicating a typical nuclear p65 positive cell. Scale bar indicates 100 µm. (C) The proportion of nuclear p65 positive nonparenchymal cells. ***P<0.001, compared with samples from the first day of infection (day 0).

To analyze the alteration of activity of NF-κB signaling in the progression of hepatic schistosomiasis, we first detected the expression and distribution of p65 (a crucial member of the NF-κB transcription family) in the liver tissues of mice by ISH. The ISH results showed that p65 signals were primarily located in the nonparenchymal cells (NPCs) and infiltrated immune cells of liver sections (Fig. 2A, 2B). Nuclear staining of p65 is used to reveal the nuclear translocation of NF-κB. Hence, to further investigate the activity of NF-κB signaling, we calculated the percent of nuclear positive cells in the NPCs. Our results revealed that NF-κB signaling was activated from 21 days post-infection (Fig. 2C).

### The activity of NF-κB signaling in HSCs post-infection

**Figure 3:**
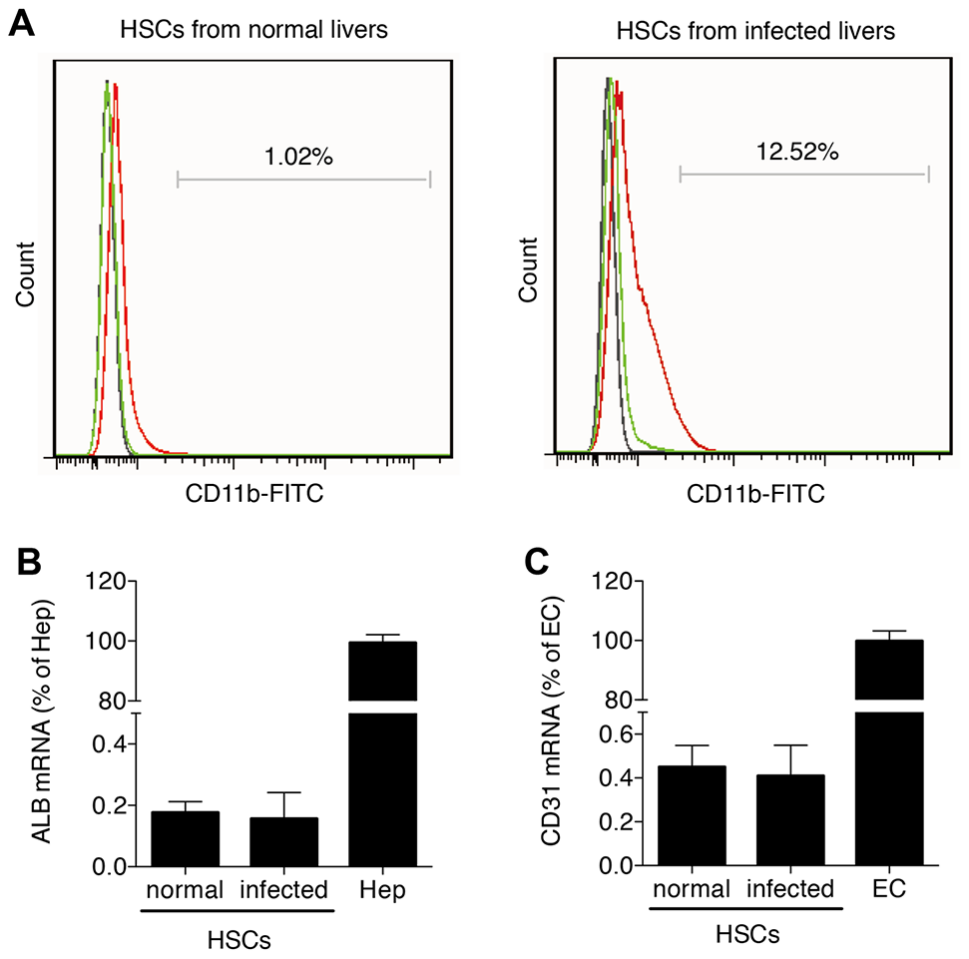
Detection of the purity of primary HSCs. Primary HSCs were isolated from three to five uninfected mice and from infected mice on day 42 post-infection. (A) Flow cytometric analysis of HSCs from uninfected and infected mice using FITC conjugated CD11b mAb compared with a concentration-matched IgG isotype control. HSCs before Kupffer cell depletion are shown in green, while HSCs after Kupffer cell depletion are shown in red. The control is shown in black. Contamination of Kupffer cell-depleted HSCs with hepatocytes (B) and endothelial cells (C) was analyzed by real-time PCR for ALB and CD31, respectively. Results were normalized to GAPDH and are expressed as mean fold ± standard deviation (SD) and compared with primary hepatocytes (“Hep” = 100%) and primary hepatic endothelial cells (“EC” = 100%).

The analysis of NF-κB signaling activity in HSCs required the use of HSCs of very high purity. Isolation of HSCs from normal (uninfected) livers generally results in >95% purity using traditional collagenase-based density centrifugation [27]; however, in HSCs isolated from infected livers we detected a moderate degree of Kupffer cell contamination (Fig. 3A), a problem previously reported [28]. Hence, we employed MACS-based Kupffer cell depletion after density centrifugation. Flow cytometric analysis revealed that CD11b-based Kupffer cell depletion strongly decreased the macrophage marker CD11b (Fig. 3A). Subsequently, we used real-time PCR analysis to detect the hepatocyte marker ALB and the hepatic endothelial cell marker CD31. We found less than 0.2% hepatocyte contamination and less than 0.5% endothelial cell contamination in HSCs isolated from the uninfected or infected mice.

**Figure 4:**
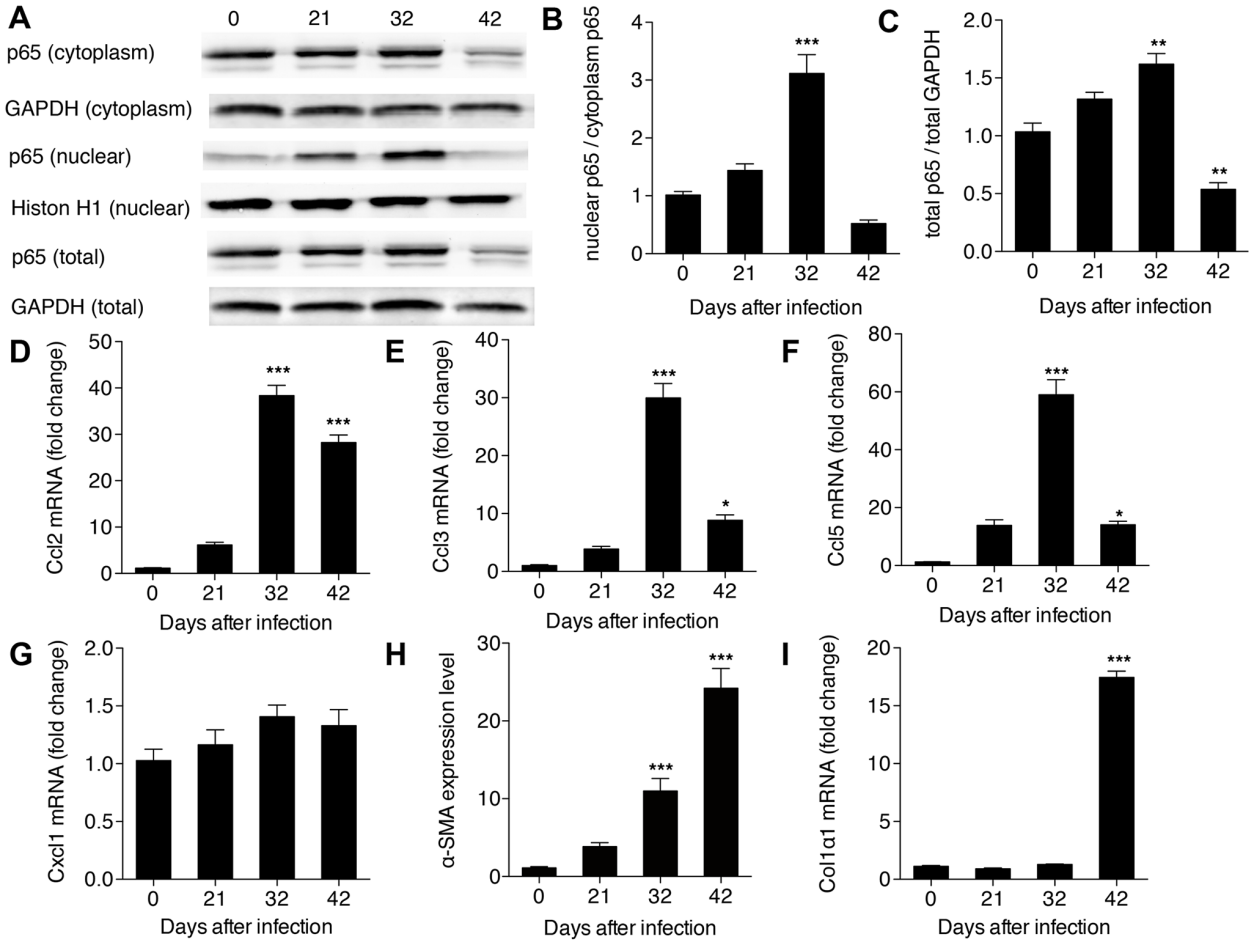
Activation of NF-κB signaling in HSCs in hepatic schistosomiasis. (A) Total protein, cytoplasm protein and nuclear protein were extracted from HSCs and then levels of p65 were detected in each by western bolt; (B) the activity of NF-κB presented by the ratio of nuclear p65 to cytoplasm p65; and (C) the total p65 level. Expression level of (D) Ccl2; (E) Ccl3; (F) Ccl5; (G) Cxcl1; (H) α-SMA; and (I) Colα1 determined from total RNA by real-time PCR. *P<0.05, **P<0.01, ***P<0.001, compared with samples from the first day of infection (day 0).

Our results revealed that NF-κB activity (presented as the ratio of nuclear p65 to cytoplasmic p65) in HSCs increased significantly after 32 days post-infection, but soon returned to pre-infection levels (Fig. 4A, 4B). In contrast, expression levels of total p65 were up-regulated over the same time period, but then down-regulated after 42 days post-infection (Fig. 4A, 4C). Consistent with the trend of NF-κB activity, the expression levels of three of the four selected chemokines regulated by NF-κB (Ccl2, Ccl3, Ccl5, but not Cxcl1), peaked on day 32 post-infection (Fig. 4D, 4E, 4F, 4G). We also used real-time PCR to detect two additional markers reflecting activation of the HSCs, α-SMA and Colα1. We found that the expression level of α-SMA was significantly up-regulated at 32 days post-infection (Fig. 4H), while the expression level of Colα1 remained unchanged until 42 days post-infection (Fig. 4I).

### TLR4 signaling in HSCs post-infection

**Figure 5:**
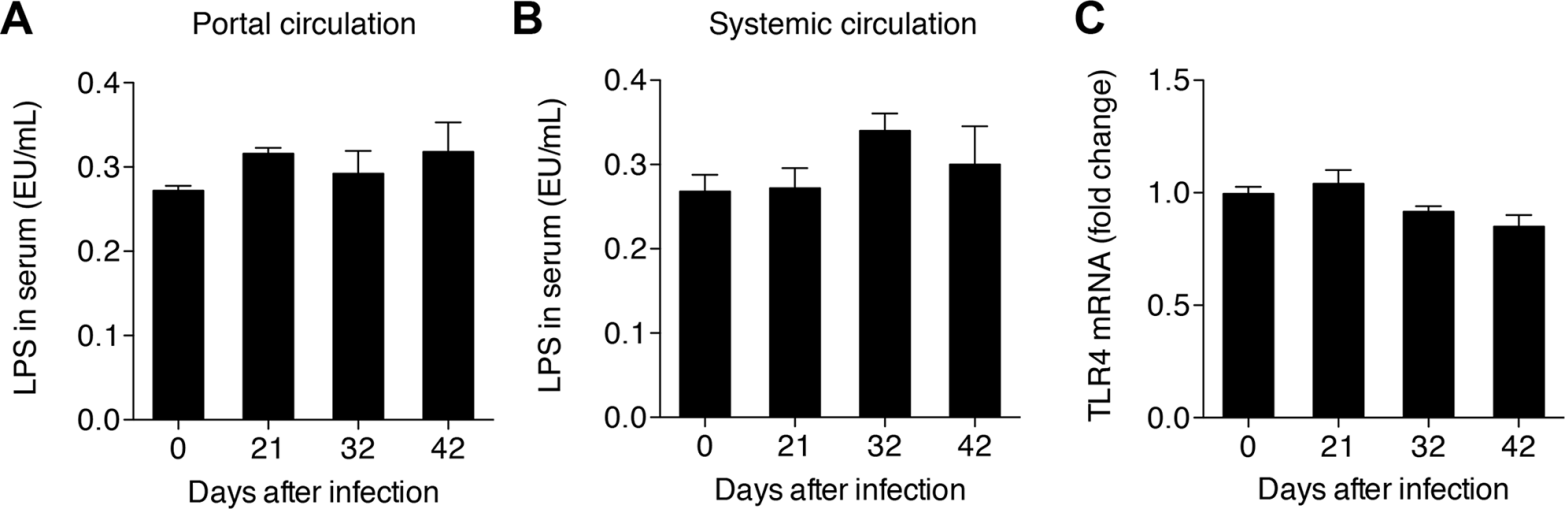
Analysis of TLR4 signaling in HSCs post-infection. LPS levels in the serum from (A) portal or (B) systemic circulation, and (C) TLR4 levels in the HSCs.

TLR4 acts as receptor for LPS, a cell-wall component of Gram-negative bacteria, one of the strongest known inducers of inflammatory processes, including the activation of NF-κB [29]. Studies have shown that when the liver is injured levels of LPS increase in the portal and systemic circulation as a result of damage to the permeability of the intestinal mucosa [30]. To validate whether the activation of NF-κB in HSCs after infection was due to the LPS stimulation through TLR4 signaling, we detected the LPS levels in the portal and systemic circulation and TLR4 expression levels in HSCs after infection. However, we found that the LPS levels in portal and systemic circulation, as well as levels of TLR4 in HSCs remained unchanged following infection with schistosoma (Fig. 5).

### MiR-146 expression in HSCs post-infection

**Figure 6:**
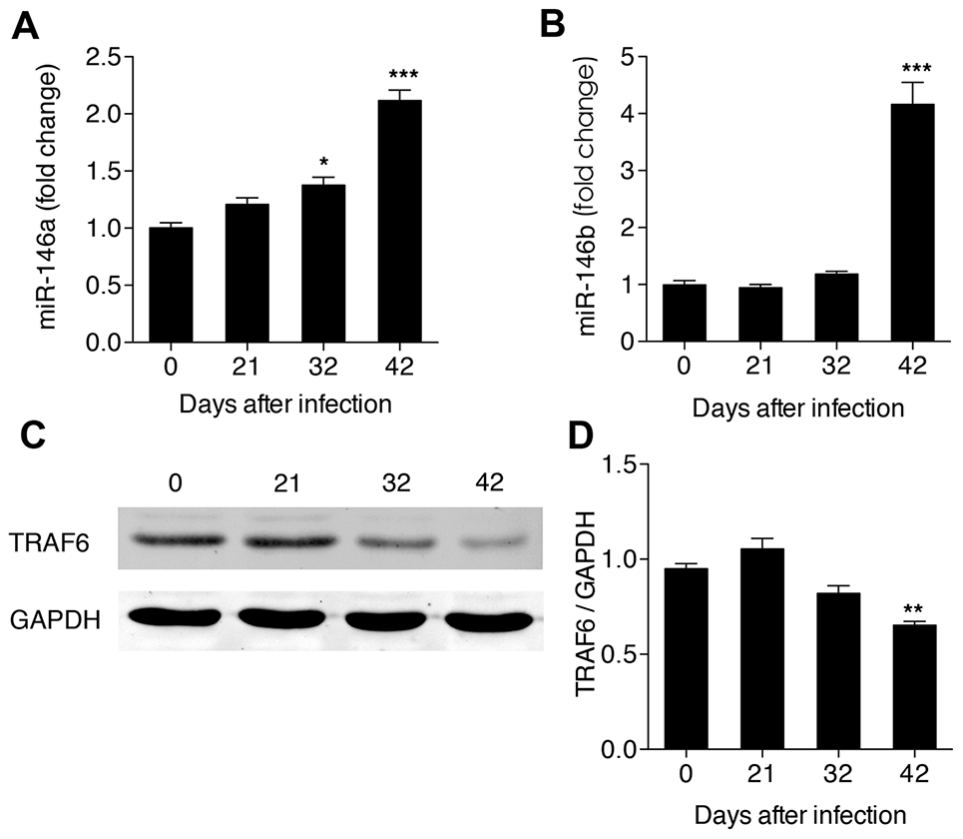
Expression levels of miRNA-146 in HSCs in hepatic schistosomiasis. Expression levels of (A) miR-146a and (B) miR-146b in total RNAs detected by real-time PCR, and (C, D) Band pattern and TRAF6 levels in total protein detected the by western blot. *P<0.05, **P<0.01, ***P<0.001, compared with samples from the first day of infection (day 0).

Studies have shown that miR-146 is a negative regulator of NF-κB signaling through its effects on IRAK1 and TRAF6, two key adapter molecules in the TLR4/NF-κB pathway [31]. Furthermore, miR-146 was significantly up-regulated in the livers of mice infected by *S. japonicum* [21]. In this study, using real-time PCR, we showed that miR-146a began to increase on day 32 post-infection, and increasing greatly on day 42 post-infection (Fig. 6A). Meanwhile, we found that miR-146b was only up-regulated significantly on day 42 post-infection (Fig. 6B). By contrast, levels of TRAF6 did not differ significantly from pre-infection levels until 42 days post-infection, when they were significantly down-regulated (Fig. 6C).

## Discussion

Hepatic schistosomiasis is a debilitating disease of human in which schistosome eggs become lodged in the host liver, leading to the development of granulomas and fibrosis. Hepatic stellate cells (HSCs) are the main sources of collagen in the liver and play a crucial role in schistosome-induced fibrogenesis. However, the role of HSCs in the inflammatory response during hepatic schistosomiasis is poorly understood. In this study, we demonstrate that NF-κB (a crucial mediator of inflammatory response) in HSCs was significantly activated at the early stage of infection in a well-studied murine model of schistosomiasis. In addition, we show that the expression levels of several chemokines that are regulated by NF-κB also exhibit a similar trend in activity. Importantly, our data revealed that miR-146 appeared to be an important negative regulator of NF-κB signaling in HSCs, and acts by targeting TRAF6.

In normal liver tissue, quiescent HSCs store vitamin A. However, in response to liver damage, they are activated and transdifferentiate into myofibroblasts, characterized by the production of ECM components rich in fibrillar collagens [2]. Studies have shown that activated HSCs are located at the peripheral of egg granulomas, and play a major role in ECM remodeling in hepatic schistosomiasis [32]. Consistent with these previous studies, we found that HSCs begin to express the marker of myofibroblasts, α-SMA, at early stages of infection (i.e. 32 days post-infection), with collagens being expressed later, when ECM begins to be deposited in the liver tissue (i.e. 42 days post-infection).

Recently, Seki *et al.* reported that in other human liver diseases, HSCs play an important role in linking hepatic inflammation to fibrogenesis via the activation of NF-κB [6]. Similarly, in this study, we found that NF-κB was significantly activated in HSCs, though only at the early stage of infection (i.e. 32 days post-infection). The pathology of schistosome infection in the liver appears tightly connected to the action of chemokines (like Ccl2), which control the migration of immune cells to the site of infection [33], [34], and mediate the chemotaxis of HSCs and macrophages [35]. In this study, we found that changes in the expression levels of several chemokines (Ccl2, Ccl3 and Ccl5) mirrored those of NF-κB signaling (similarly peaking 32 days post-infection). These results combined indicate that HSCs are involved in schistosome-induced hepatic inflammation in the murine model of schistosomiasis, and might operate via NF-κB signaling.

In other murine models of hepatic fibrosis, increased level of LPS in the portal circulation induce activation of NF-κB in HSCs by binding to TLR4, the expression of which is also up-regulated in this process [6]. However, in the murine model of hepatic schistosomiasis used in this study, LPS levels in both portal and systemic circulation remained unchanged following infection, as did expression levels of the receptor, TLR4. These results suggest that the regulation of NF-κB activation varies with the distinct pathogenesis of liver damage. It is worthy to point out that Anthony *et al.* recently reported that *S. japonicum* eggs induced HSCs to express high levels of pro-inflammatory cytokines which linked to NF-κB activation, but blocked the response of HSCs to TGF-β, the most potent fibrogenic activator of these cells [8]. Their study appeared to contradict with a previous study which demonstrated LPS induced TLR-4 stimulation in HSCs renders these cells expressing high levels of pro-inflammatory cytokines and hypersensitive to TGF-β by NF-κB activation [6]. Our study could explain this contradiction, that is, the pathway of NF-κB activation in HSCs during hepatic schistosomiasis might not be through TLR-4 signaling. However, whether *S. japonicum* eggs can directly activate NF-κB signaling in HSCs is still elusive.

MiRNA are small noncoding RNAs that control the translation and transcription of many genes. Recent studies have revealed the importance of several miRNAs, especially the miR-146, in the regulation of NF-κB signaling [17]. Taganov *et al.* have proposed a model of miR-146 negative feedback regulation of NF-κB signaling in which the activated NF-κB pathway leads to induction of miR-146 expression, which targets IRAK1 and TRAF6 and results in the attenuation of NF-κB signaling [31]. We hypothesize that there was a similar negative feedback in the progression of hepatic schistosomiasis, that is, NF-κB signaling in HSCs was activated at the early stage of infection and the expression of miR-146a/b up-regulated concurrently, subsequently targeting TRAF6 and, eventually, sequestering the NF-κB signaling. In addition, we speculate that as the typical granulomas begin to form only on day 42 post-infection, by which time NF-κB signaling has been attenuated, miR-146 appears to be a critical negative regulator of the granulomatous process that operates via targeting NF-κB signaling.

Taken together, the results of our study indicate that HSCs play an important role in the progression of hepatic schistosomiasis by linking liver inflammation to fibrosis via NF-κB signaling, and suggest that miR-146 appears to be a critical for regulating this process. These findings are significant and imply that manipulating the function of HSCs by targeting either NF-κB signaling or miR-146 expression may provide a novel method of treating hepatic schistosomiasis.
