# Correction: Activation of Nuclear Factor Kappa B in the Hepatic Stellate Cells of Mice with Schistosomiasis Japonica

**DOI:** 10.1371/journal.pone.0243667

**Published:** 2020-12-17

**Authors:** Xing He, Guangbin Pu, Rui Tang, Dongmei Zhang, Weiqing Pan

After this article [1] was published, it was noted that the p65 (cytoplasm) and p65 (total) panels in Fig 4A report the same data. The authors apologize for the error and noted that the wrong data were included in the p65 (cytoplasm) panel. An updated Fig 4 is provided here and the underlying data for this figure are in S1 File. The image issues do not affect the quantitative results in Fig 4B and 4C.

**Fig 4:**
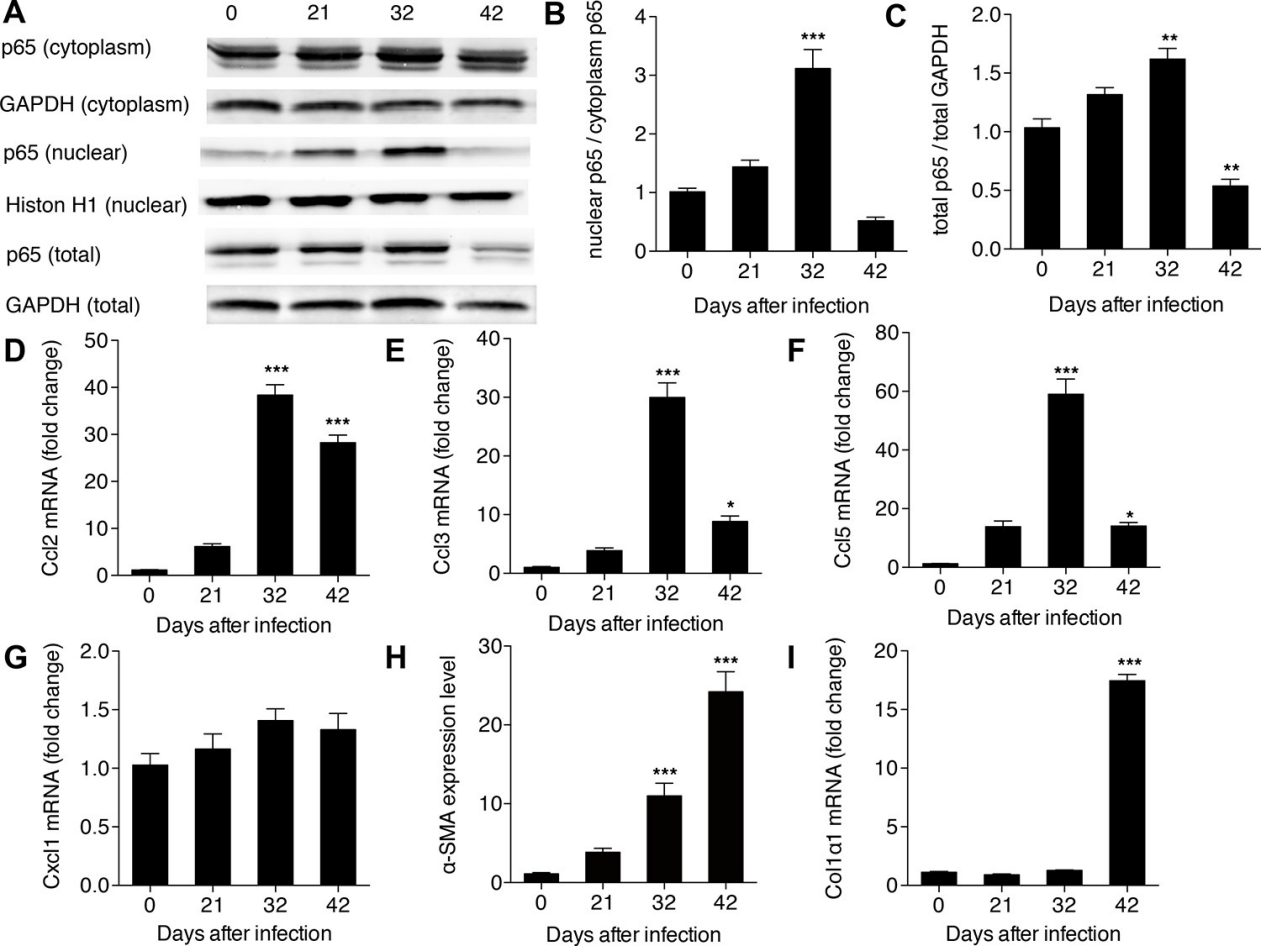
Activation of NF-κB signaling in HSCs in hepatic schistosomiasis. (A) Total protein, cytoplasm protein and nuclear protein were extracted from HSCs and then levels of p65 were detected in each by western bolt; (B) the activity of NF-κB presented by the ratio of nuclear p65 to cytoplasm p65; and (C) the total p65 level. Expression level of (D) Ccl2; (E) Ccl3; (F) Ccl5; (G) Cxcl1; (H) α-SMA; and (I) Colα1 determined from total RNA by real-time PCR. *P<0.05, **P<0.01, ***P<0.001, compared with samples from the first day of infection (day 0).

The Results section text discussing Fig 4A–4C are revised as follows:

Our results revealed that NF-κB activity (presented as the ratio of nuclear p65 to cytoplasmic p65) in HSCs increased significantly by 32 days post-infection (dpi), but returned to pre-infection levels by 42 dpi (Fig 4A and 4B). In contrast, expression levels of total p65 increased between 0 and 32 dpi, and then were down-regulated by 42 dpi to levels below those observed at baseline (Fig 4A and 4C).

The original flow cytometry data files underlying Fig 3A are no longer available. The underlying data to support other results in the article are provided in S2-S4 Files.

## Supporting information

S1 File Original data underlying results reported in Fig 4A. (PPT)

S2 File Raw quantitative data underlying results reported in Figs 1–6. (ZIP)

S3 File Original image data underlying results reported in Figs 1A, 2A and 6C. (ZIP)
