# Supplementary material for: Correction: Activation of Nuclear Factor Kappa B in the Hepatic Stellate Cells of Mice with Schistosomiasis Japonica
Source: PLoS One. 2020 Dec 17;15(12):e0243667. doi: 10.1371/journal.pone.0243667 (PMC7746179; doi:10.1371/journal.pone.0243667)
Supplement: S1 File — (PPT) [file pone.0243667.s001.ppt]

## Slide 1
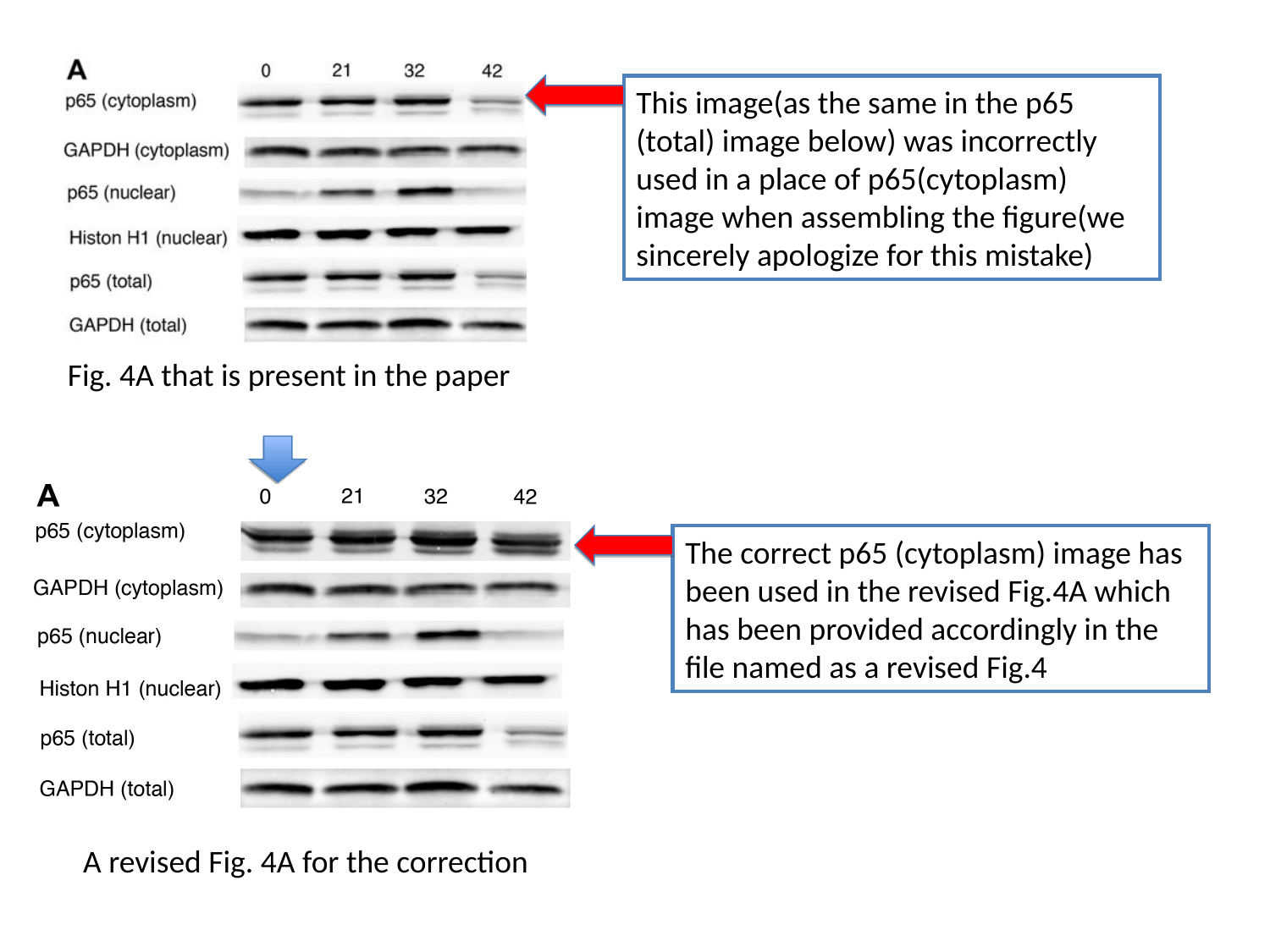

This image(as the same in the p65 (total) image below) was incorrectly used in a place of p65(cytoplasm) image when assembling the figure(we sincerely apologize for this mistake)
Fig. 4A that is present in the paper
The correct p65 (cytoplasm) image has been used in the revised Fig.4A which has been provided accordingly in the file named as a revised Fig.4
A revised Fig. 4A for the correction

## Slide 2
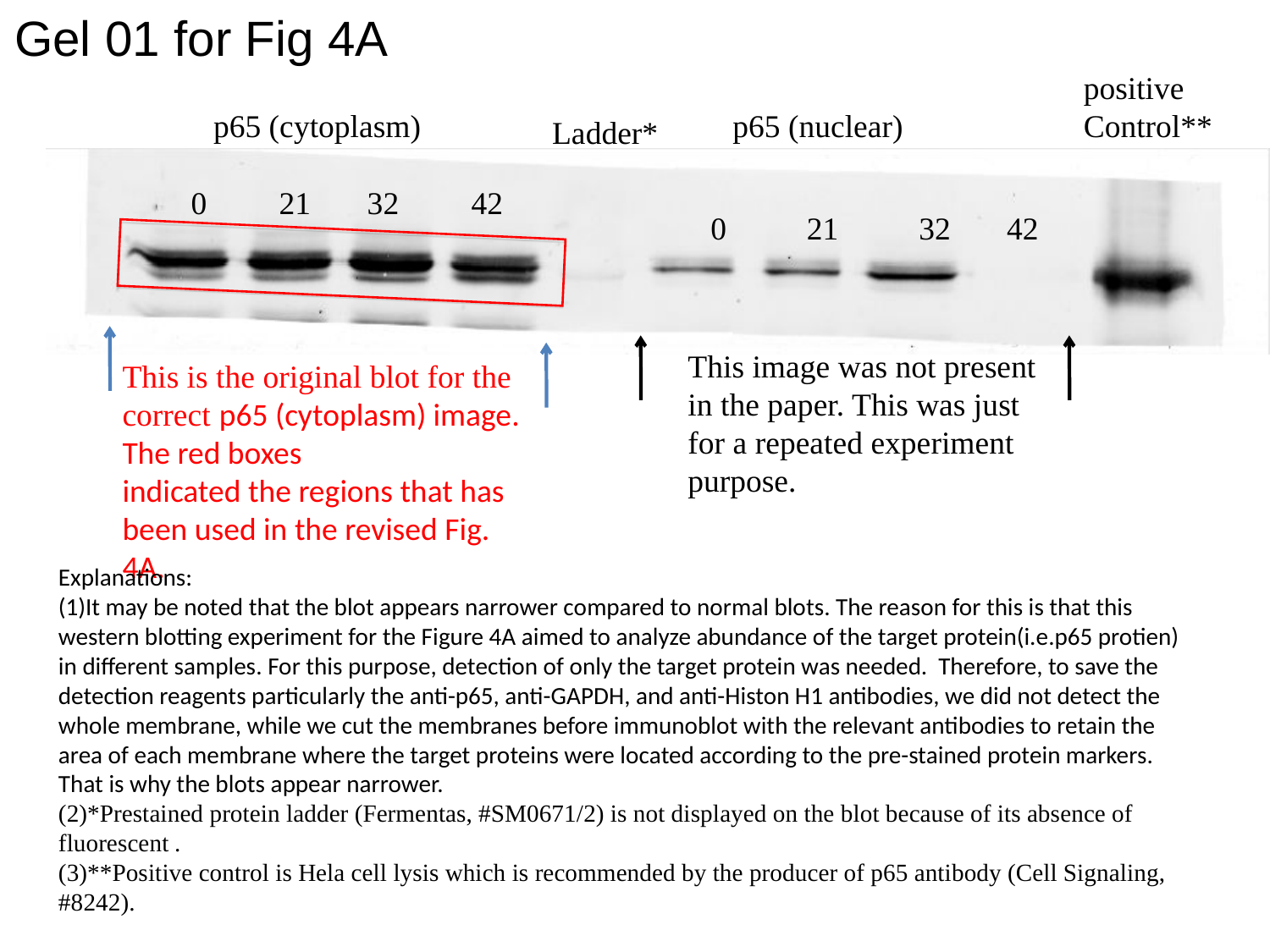

Gel 01 for Fig 4A
positive
Control**
p65 (cytoplasm)
p65 (nuclear)
Ladder*
0 21 32 42
0 21 32 42
This image was not present in the paper. This was just for a repeated experiment purpose.
This is the original blot for the correct p65 (cytoplasm) image. The red boxes
indicated the regions that has been used in the revised Fig. 4A.
Explanations:
(1)It may be noted that the blot appears narrower compared to normal blots. The reason for this is that this western blotting experiment for the Figure 4A aimed to analyze abundance of the target protein(i.e.p65 protien) in different samples. For this purpose, detection of only the target protein was needed. Therefore, to save the detection reagents particularly the anti-p65, anti-GAPDH, and anti-Histon H1 antibodies, we did not detect the whole membrane, while we cut the membranes before immunoblot with the relevant antibodies to retain the area of each membrane where the target proteins were located according to the pre-stained protein markers. That is why the blots appear narrower.
(2)*Prestained protein ladder (Fermentas, #SM0671/2) is not displayed on the blot because of its absence of fluorescent .
(3)**Positive control is Hela cell lysis which is recommended by the producer of p65 antibody (Cell Signaling, #8242).

## Slide 3
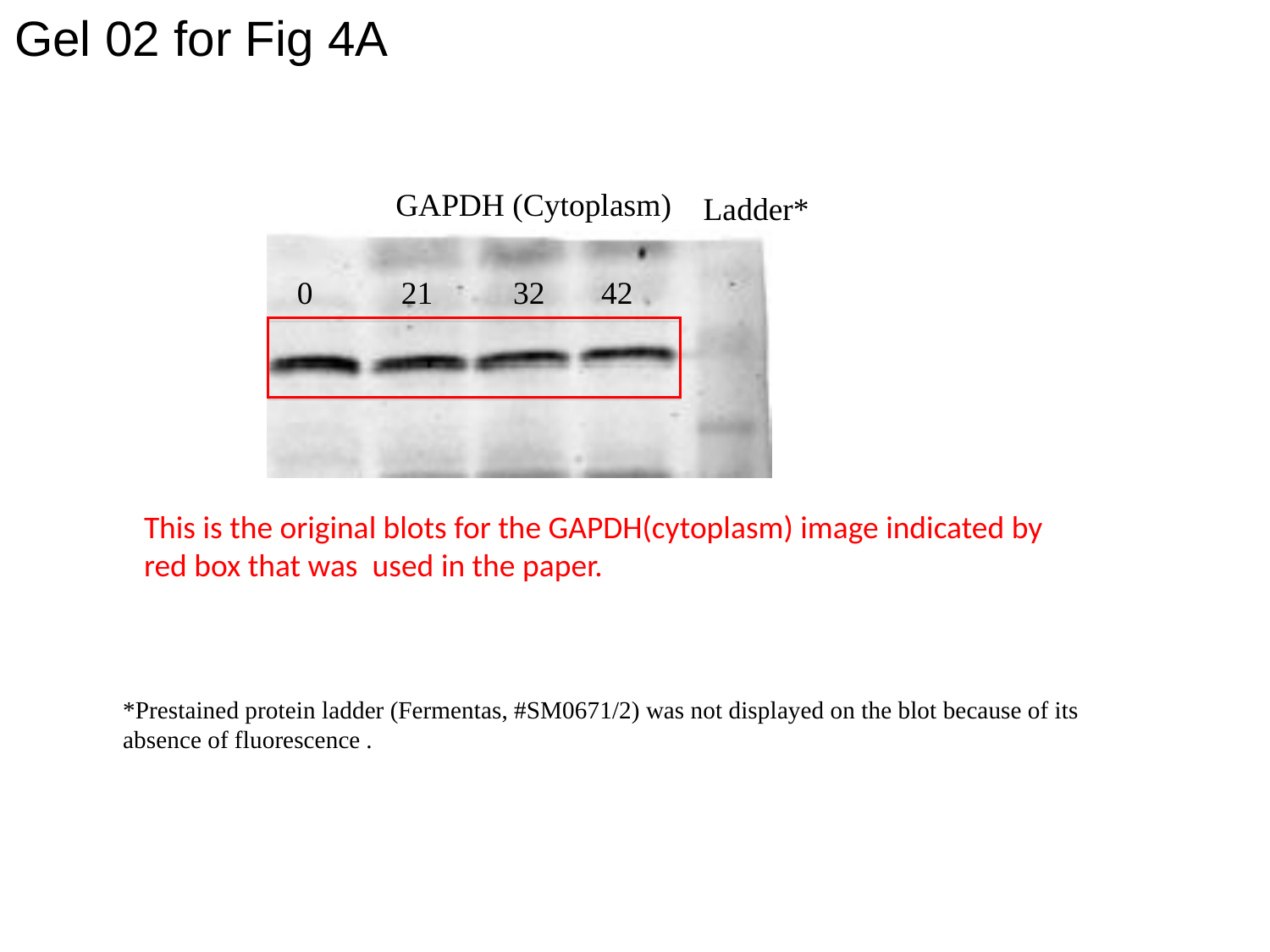

Gel 02 for Fig 4A
GAPDH (Cytoplasm)
Ladder*
0 21 32 42
This is the original blots for the GAPDH(cytoplasm) image indicated by red box that was used in the paper.
*Prestained protein ladder (Fermentas, #SM0671/2) was not displayed on the blot because of its absence of fluorescence .

## Slide 4
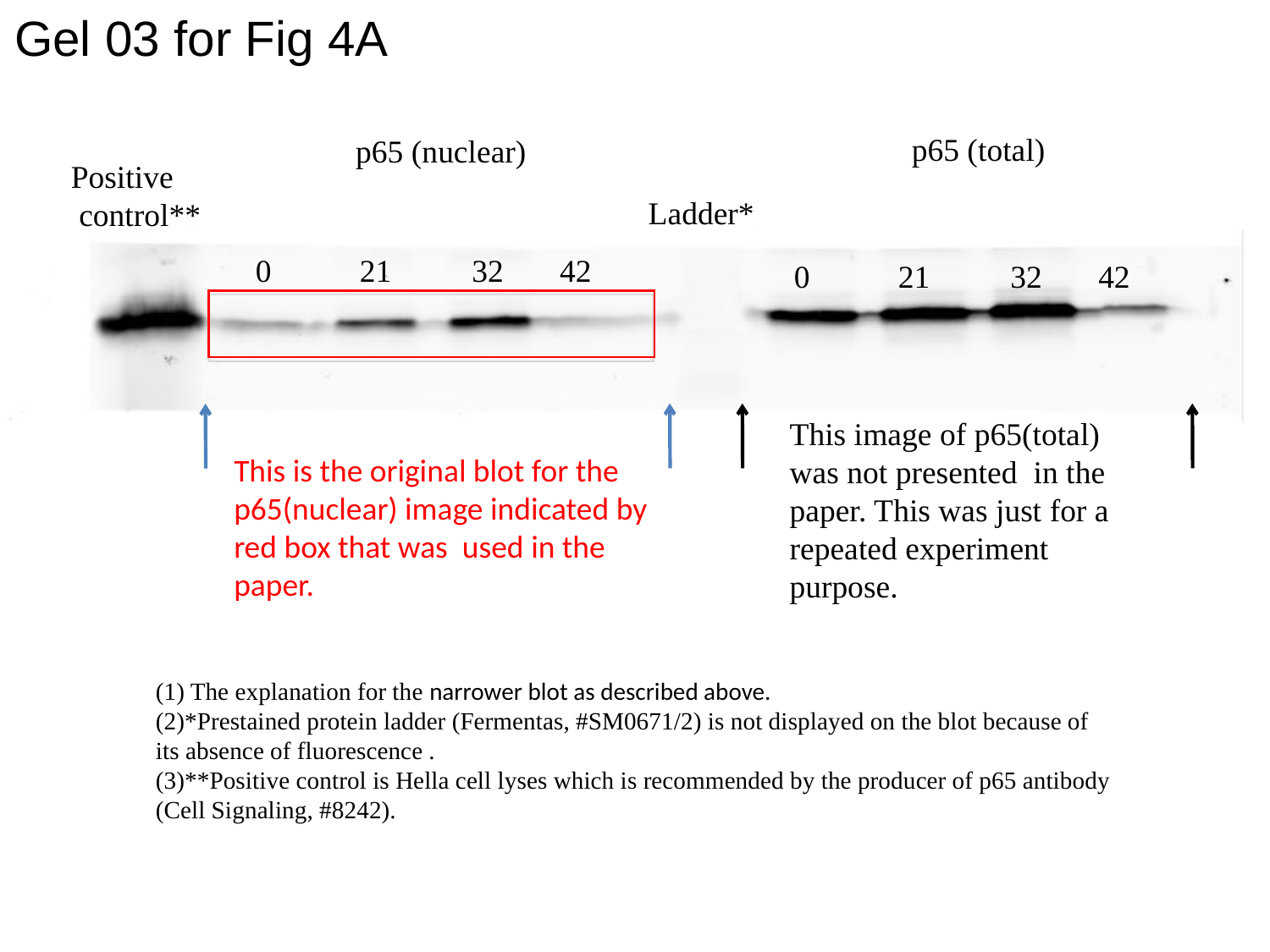

Gel 03 for Fig 4A
p65 (total)
p65 (nuclear)
Positive
 control**
Ladder*
0 21 32 42
0 21 32 42
This image of p65(total) was not presented in the paper. This was just for a repeated experiment purpose.
This is the original blot for the p65(nuclear) image indicated by red box that was used in the paper.
(1) The explanation for the narrower blot as described above.
(2)*Prestained protein ladder (Fermentas, #SM0671/2) is not displayed on the blot because of its absence of fluorescence .
(3)**Positive control is Hella cell lyses which is recommended by the producer of p65 antibody (Cell Signaling, #8242).

## Slide 5
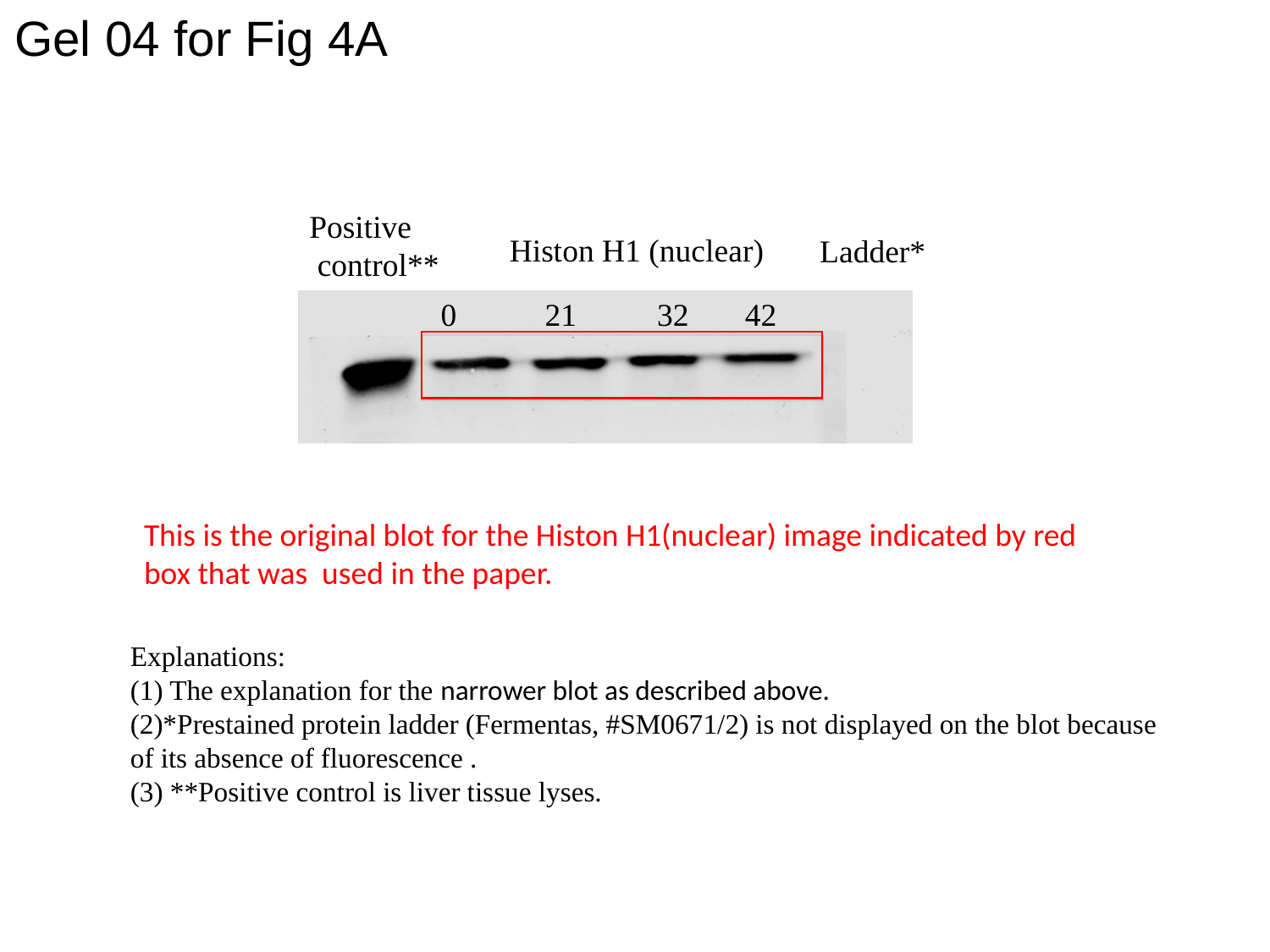

Gel 04 for Fig 4A
Positive
 control**
Histon H1 (nuclear)
Ladder*
0 21 32 42
This is the original blot for the Histon H1(nuclear) image indicated by red box that was used in the paper.
Explanations:
(1) The explanation for the narrower blot as described above.
(2)*Prestained protein ladder (Fermentas, #SM0671/2) is not displayed on the blot because of its absence of fluorescence .
(3) **Positive control is liver tissue lyses.

## Slide 6
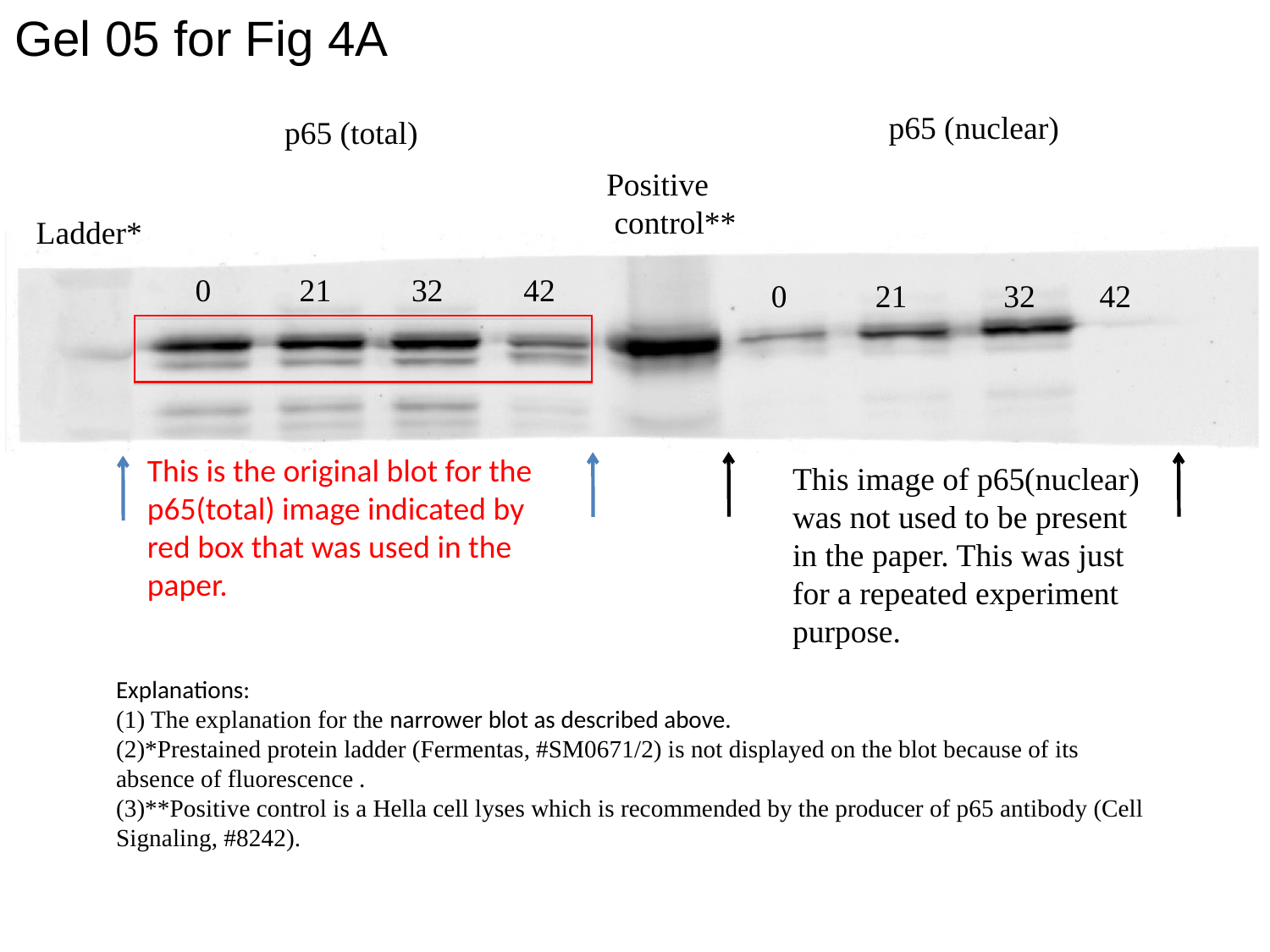

Gel 05 for Fig 4A
p65 (nuclear)
p65 (total)
Positive
 control**
Ladder*
0 21 32 42
0 21 32 42
This is the original blot for the p65(total) image indicated by red box that was used in the paper.
This image of p65(nuclear) was not used to be present in the paper. This was just for a repeated experiment purpose.
Explanations:
(1) The explanation for the narrower blot as described above.
(2)*Prestained protein ladder (Fermentas, #SM0671/2) is not displayed on the blot because of its absence of fluorescence .
(3)**Positive control is a Hella cell lyses which is recommended by the producer of p65 antibody (Cell Signaling, #8242).

## Slide 7
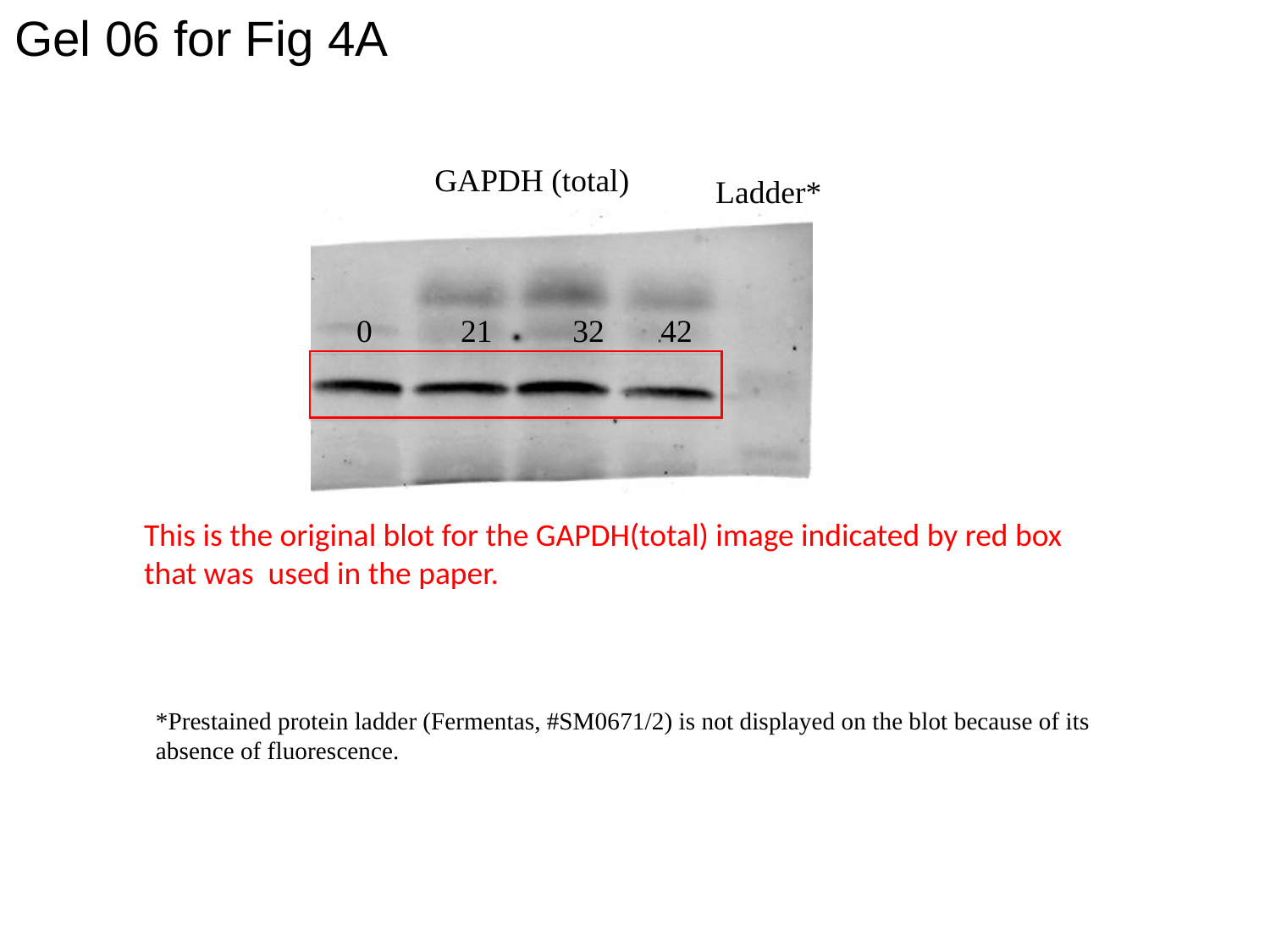

Gel 06 for Fig 4A
GAPDH (total)
Ladder*
0 21 32 42
This is the original blot for the GAPDH(total) image indicated by red box that was used in the paper.
*Prestained protein ladder (Fermentas, #SM0671/2) is not displayed on the blot because of its absence of fluorescence.
